# Supplementary material for: Stacking Model‐Based Classifiers for Dealing With Multiple Sets of Noisy Labels
Source: Biom J. 2025 Mar 12;67(2):e70042. doi: 10.1002/bimj.70042 (PMC11898607; doi:10.1002/bimj.70042)
Supplement: Supplementary file 1 — Supporting Information [file BIMJ-67-e70042-s001.zip › density-based-ensemble-model-main/competitors_real_data/netlab-master/netlab-master/help/datread.htm]

Netlab Reference Manual datread


# datread

## Purpose

Read data from an ascii file.

## Synopsis

```
[x, t, nin, nout, ndata] = datread(filename)
```

## Description

`[x, t, nin, nout, ndata] = datread(filename)` reads from
the file `filename` and returns a matrix `x` of input vectors,
a matrix `t` of target vectors, and integers `nin`, `nout`
and `ndata` specifying the number of inputs, the number of outputs
and the number of data points respectively.

The format of the data file is as follows: the first row contains the
string `nin` followed by the number of inputs, the second row
contains the string `nout` followed by the number of outputs, and
the third row contains the string `ndata` followed by the number
of data vectors. Subsequent lines each contain one input vector
followed by one output vector, with individual values separated by
spaces.

## Example

For the XOR data set we have

## See Also

`nin2nout1ndata40.000000e+000.000000e+001.000000e+000.000000e+001.000000e+000.000000e+001.000000e+000.000000e+000.000000e+001.000000e+001.000000e+001.000000e+00SeeAlsodatwrite`

---

**Pages:**
Index


---

Copyright (c) Ian T Nabney (1996-9)
